# Circulating Platelet-Derived Microparticles Associated with Postdischarge Major Adverse Cardiac Events in ST-Elevation Acute Myocardial Infarction

**DOI:** 10.1155/2020/6721584

**Published:** 2020-07-06

**Authors:** Anggoro Budi Hartopo, Dyah Samti Mayasari, Ira Puspitawati, Hasanah Mumpuni

**Affiliations:** ^1^Department of Cardiology and Vascular Medicine, Faculty of Medicine, Public Health and Nursing, Universitas Gadjah Mada–Dr. Sardjito Hospital, Yogyakarta, Indonesia; ^2^Department of Clinical Pathology and Laboratory Medicine, Faculty of Medicine, Public Health and Nursing, Universitas Gadjah Mada–Dr. Sardjito Hospital, Yogyakarta, Indonesia

## Abstract

**Introduction:**

Platelet-derived microparticles (PDMPs) measurement adds prognostic implication for ST-elevation acute myocardial infarction (STEMI). The long-term implication of PDMPs in STEMI needs to be corroborated.

**Methods:**

The research design was a cohort study. Subjects were STEMI patients and were enrolled consecutively. The PDMPs were defined as microparticles bearing CD41(+) and CD62P(+) markers detected with flow cytometry. The PDMPs were measured on hospital admission and 30 days after discharge. The outcomes were major adverse cardiac events (MACE), i.e., a composite of cardiac death, heart failure, cardiogenic shock, reinfarction, and resuscitated ventricular arrhythmia, occurring from hospitalization until 1 year after discharge.

**Results:**

We enrolled 101 subjects with STEMI. During hospitalization, 17 subjects (16.8%) developed MACE. The PDMPs were not different between subjects with MACE and those without (median (IQR): 3305.0/*μ*L (2370.0–14690.5/*μ*L) vs. 4452.0/*μ*L (2024.3–14396.8/*μ*L), *p*=0.874). Forty-five subjects had increased PDMPs in 30 days after discharge as compared with on-admission measurement. Subjects with increased PDMPs had significantly higher 30-day MACE as compared to subjects with decreased PDMPs 17 (37.8%) vs. 6 (16.7%, *p*=0.036). There was a trend toward higher MACE in subjects with increased PDMPs as compared to those with decreased PDMPs in 90 days after discharge (48.9% vs. 30.6%, *p*=0.095) and 1 year after discharge (48.9% vs. 36.1%, *p*=0.249).

**Conclusion:**

The PDMPs level was increased from the day of admission to 30 days after discharge in patients with STEMI. The persistent increase in the PDMPs level in 30 days after the STEMI event was associated with the 30-day postdischarge MACE and trended toward increased MACE during the 90-day and 1-year follow-up.

## 1. Introduction

The majority of ST-elevation acute myocardial infarction (STEMI) reflects a total obstruction of major infarct-related coronary arteries due to ruptured atherosclerotic plaque and subsequent occluded thrombus [1]. The myocardial infarction risk can be prevented from irreversible damage by the rapidity of reperfusion and timely medical management [1]. The management approaches of STEMI with coronary reperfusion, both fibrinolysis and primary percutaneous coronary intervention (PPCI), and pharmacology therapy have successfully overcome the occurrence of in-hospital major adverse cardiac events (MACE) [2]. However, the deformed myocardia will undergo remodeling, which, in the long term, can impair the heart function and lead to left heart failure [1].

The role of platelet activation in the exaggeration of a developing thrombus after ruptured plaque has been demonstrated. Platelet-derived microparticles (PDMPs) are released by a platelet upon activation [3]. In STEMI, the increased level of PDMPs has been corroborated as compared with other types of myocardial infarction and associated with damaged myocardia affected by STEMI [4–6]. In the short-term complications of STEMI, PDMPs increased in subjects with cardiogenic shock and mortality [7]. In a retrospective study, the long-term observation showed that the PDMPs level in the acute phase of STEMI was associated with cardiovascular mortality although not with MACE [8]. Furthermore, an increased PDMPs level measured after acute STEMI events were still associated with acute heart failure during an acute myocardial infarction episode [9].

## 2. Aim

This study aimed to corroborate previous studies by performing a prospective study and measuring the PDMPs level to demonstrate the prognostic role of PDMPs in the long-term follow-up after acute STEMI.

## 3. Methods

### 3.1. Research Design and Subjects

The research design was a cohort study. We conducted a consecutive sampling to enroll subjects diagnosed as STEMI. We enrolled subjects admitted to the emergency unit and managed in the intensive cardiac care unit (ICCU) of Dr. Sardjito Hospital, Yogyakarta, Indonesia. The research was conducted from January 2017–January 2019. The STEMI was diagnosed based on the criteria of symptoms of chest pain, specific electrocardiogram criteria, and/or elevated hs-troponin I markers [10].

The inclusion criteria for subjects' enrollment were as follows: (1) diagnosed with STEMI, (2) male or female patients, (3) age between 35 and 75 years, (4) the onset of chest pain ≤24 hours, and (5) no previous fibrinolytic or heparin treatment before admission. The exclusion criteria were as follows: (1) previously known chronic heart failure (NYHA class ≥II), chronic kidney disease (stage IV-V), hepatic cirrhosis, and chronic inflammatory diseases (such as chronic arthritis, psoriasis, and inflammatory bowel disease), (2) previously known malignancy, (3) concomitant severe acute infection, sepsis, and acute stroke during hospital care, and (4) thrombocytopenia during hospital care. During observation, subjects signed an informed consent form to participate in the study. The study was approved by the Medical and Health Research Ethics committee of the Faculty of Medicine, Public Health, and Nursing, Universitas Gadjah Mada and Dr. Sardjito Hospital, Yogyakarta, Indonesia.

### 3.2. Initial Examination and Procedures

The attending physician examined the subjects on admission in the emergency unit. Demography and clinical data were documented in the case report form. The attending cardiologists performed initial interventional procedures, if indicated, such as fibrinolysis, PPCI, and/or rescue PCI. The subsequent management in the ICCU was at the discretion of the attending cardiologists according to patients' clinical condition.

### 3.3. Laboratory Examination

The antecubital vein blood samples were taken on admission prior to any interventional procedures and analyzed for hematology examination with an automated blood cell counter, as well as for blood chemistry with a chemical analyzer performed in the central hospital laboratory. For microparticle examination, the blood samples were withdrawn from antecubital veins of subjects in a supine position with a 22 G needle and BD-Vacutainer tube containing a sodium citrate anticoagulant (BD Diagnostic, Maryland, USA). Two timeframes of blood sample collection were performed, within 24 hours of hospital admission and at 30 days after discharge. The samples were immediately double centrifuged at 160 g for 10 min and 6000 g for 1 min to collect platelet-poor plasma (PPP). A 50 *μ*l of PPP was incubated with FITC Anti-Human CD41 (Biolegend, San Diego, USA) and PE Anti-Human CD62P (Biolegend, San Diego, USA) in a TruCount tube (BD Diagnostic, Maryland, USA) for 30 min to detect platelet-derived microparticles (PDMPs). The samples were analyzed with a flow cytometer (FACS Calibur, Becton Dickinson, Maryland, USA). Platelet microparticles (PDMPs) were determined as particles with a diameter size < 1 *μ*m from standardized SSC- and FSC-height and a positive marker for CD41 and CD62P. The absolute number of PDMPs was calculated based on the formula of CD41 and CD62P positivity within the threshold of 1 *μ*m gated events (R3) multiplied by the total number of bead events (based on the datasheet, it was 50,000) and normalized with the number of TruCount bead events and volume of the sample [5].

### 3.4. In-Hospital Observation and Outcomes

The subjects were observed during acute intensive care in the ICCU. The end of observation was until discharge from the ICCU or a fatal cardiac event occurred. The outcome of interest was major adverse cardiac events (MACE). The MACE was a composite of cardiac death, acute heart failure, cardiogenic shock, reinfarction, and resuscitated ventricular arrhythmia (VT/VF). The MACE was assessed and managed by attending cardiologists unaware of the PDMPs measurement. A cardiac death was mortality by cardiac causes. Acute heart failure was defined based on signs and symptoms and intravenous diuretics administration. Cardiogenic shock was defined as signs and symptoms of low perfusion related to systolic blood pressure <90 mmHg and inotropics or/and vasopressor drugs administration. Reinfarction was defined as new event of anginal pain, ST-elevation electrocardiogram, and increased CKMB or troponin I in previously stable patients. Resuscitated VT/VF was defined as the return of spontaneous circulation following cardiopulmonary resuscitation and defibrillation after an episode of VT/VF. Subject selection, examination, and in-hospital observation followed the methods of Hartopo et al. [11].

### 3.5. The 30-Day, 90-Day, and 1-Year Follow-Up and Outcome

The discharged subjects were followed up until 1 year after discharge. They were asked to visit the cardiology research office (CRO) in our hospital at day 30 after discharge to have the blood sample collected and interview regarding MACE and the treatment they had. After 90 days and 1 year after discharge, the subjects were asked to visit the CRO again to have an evaluation regarding MACE and their management during the time frame. The MACE occurrence was collected during these timeframes, which included a composite of cardiac death, chronic heart failure, and hospital readmission of any cardiac causes. The examiners were blind to the PDMPs level of the subjects.

### 3.6. Statistical Analysis

The subjects were divided into two groups based on MACE occurrences. Based on the PDMPs level difference at the day of admission and 30 days after discharge, the subjects were divided into two groups, subjects with increased PDMPs and subjects with decreased PDMPs. Variable comparison between the two groups was performed with Student's *T*-test or the Mann–Whitney *U* test for continuous variables and the chi-square test or Fisher exact test for categorical variables. A *p* value < 0.05 was considered statistically significant.

## 4. Results

### 4.1. Clinical Characteristics and In-Hospital MACE

We enrolled 101 subjects with STEMI. Among them, 17 subjects (16.8%) developed MACE during intensive hospitalization in the ICCU. Most MACE were acute heart failure (14 subjects (82.6%), and others were cardiogenic shock (2 subjects, 11.6%) and resuscitated ventricular tachycardia (1 subjects, 5.8%). There were no significant differences in the gender, age, cardiovascular disease risk factors, onset of angina, and infarct location between subjects with MACE and those without. Subjects with MACE had a Killip class ≥2 and a significantly increased heart rate. The revascularization procedures as the initial treatment, both primary PCI and fibrinolysis, were performed in 85 subjects (84.2%). There were no differences in the type of revascularization, vessel disease, culprit lesion, and intervention between subjects with MACE and those without. The serum creatinine level was significantly increased in subjects with MACE, whereas other laboratory results were comparable. The use of intravenous furosemide was higher in subjects with MACE.  Table 1 shows the clinical characteristics of subjects based on MACE occurrences.

### 4.2. PDMPs Level and In-Hospital MACE

**Figure 1:**
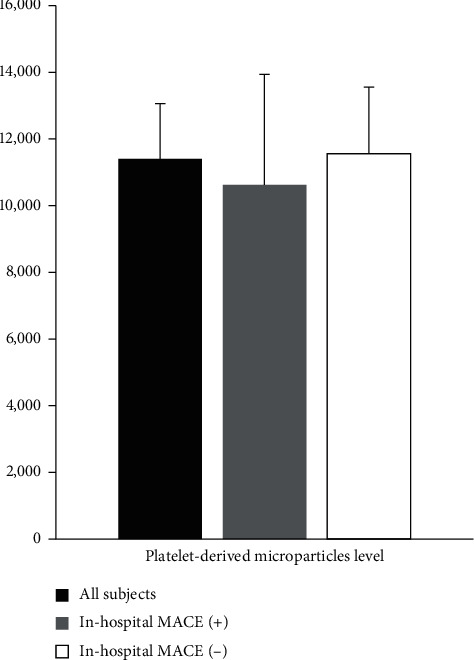
Platelet-derived microparticles (PDMPs) level in all subjects with STEMI (black bar), subjects with in-hospital MACE (grey bar), and subjects without in-hospital MACE (white bar) (mean ± SEM: 11401.4 ± 1657.1/*μ*L, 10623.3 ± 3315.4/*μ*L, and 11558.2 ± 1883.4/*μ*L, respectively). There was a tendency toward a decreased PDMPs level in subjects with in-hospital MACE (*p*=0.874).

**Figure 2:**
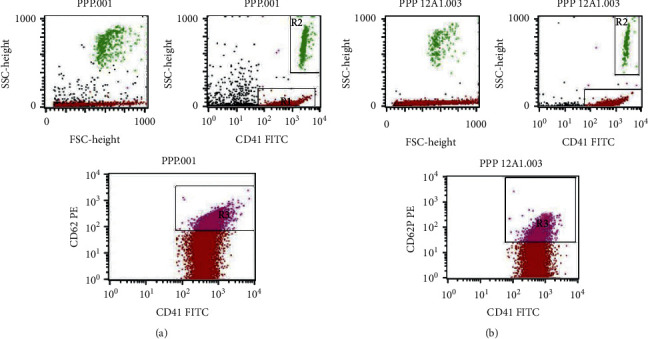
Representative result of the PDMPs level (CD41(+)/CD62P(+)) microparticles (R3) measured by FACS. (a) Representative for subjects without MACE. (b) Representative for subjects with MACE.

The median of the PDMPs level in all subjects was 4378.0/*μ*L (interquartile range (IQR): 2108.5–14052.5/*μ*L), and its mean level was 11401.4/*μ*L. The mean level with a standard error of the mean (SEM) of PDMPs between subjects with MACE and those without was not significantly different (mean ± SEM: 10623.3 ± 3315.4/*μ*L vs. 11558.9 ± 1883.4/*μ*L; median (IQR): 3305.0/*μ*L (2370.0–14690.5/*μ*L) vs. 4452.0/*μ*L (2024.3–14396.8/*μ*L), *p* = 0.874), respectively.  Figure 1 indicates there was no significant difference in the PDMPs level between subjects with MACE and those without. There was a tendency toward lower the PDMPs level in subjects with MACE.  Figure 2 indicates the representative measurement of the PDMPs level using FACS.

### 4.3. Clinical Characteristics and 30-Day MACE

At 30 days after discharge, as many as 81 subjects could be followed up and taken blood samples for PDMPs measurement. Twenty subjects (19.8%) refused to visit hospital for blood sampling collection. None of them reported cardiac death and hospital admission within 30 days. Since the PDMPs result was obtained from 81 subjects, we included 81 subjects for analysis of 30-day MACE. Among the 81 subjects, 23 subjects (28.4%) developed MACE. Most MACE were heart failure (21 subjects, 91%), followed by hospital admission of reinfarction and arrhythmia (2 subjects, 9%). There were no significant differences in the gender, age, cardiovascular disease risk factors, onset of angina, and infarct location between subjects with MACE and those without. Subjects with MACE had a Killip class ≥2 and a significantly increased heart rate on admission. There were no differences in the type of revascularization, number of vessel disease, culprit lesion, and intervention between subjects with MACE and those without. The laboratory results were comparable. The use of intravenous furosemide on admission was higher in subjects with MACE.  Table 2 shows the clinical characteristics of subjects who underwent the 30-day follow-up study.

### 4.4. PDMPs Level and 30-Day MACE

**Figure 3:**
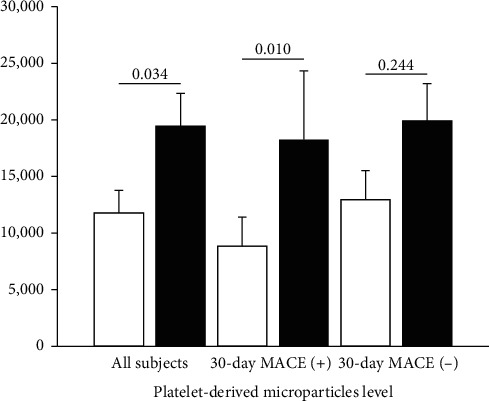
Platelet-derived microparticles (PDMPs) level in all subjects with STEMI, subjects with 30-day MACE, and subjects without 30-day MACE. There was a significant increase in the PDMPs level from admission (white bars) to 30 days after discharge (black bars) in all subjects (mean ± SEM: 11795.7 ± 1975.5/*μ*L vs. 19450.5 ± 2888.5/*μ*L, *p*=0.034) and subjects with 30-day MACE (mean ± SEM: 8853.4 ± 2569.5/*μ*L vs. 18263.5 ± 6056.8/*μ*L, *p*=0.010).

There was an increase in the PDMPs level from the time of admission to 30 days after discharge in all subjects ((median (IQR): 4284.0/*μ*L (1893.0–14052.5/*μ*L) vs. 9448.0/*μ*L (1575.5–28831.5/*μ*L) and (mean ± SEM: 11795.7 ± 1975.5/*μ*L vs. 19450.5 ± 2888.5/*μ*L), *p* = 0.034 (Wilcoxon signed rank test). The PDMPs level on admission between subjects with 30-day MACE and those without was not significantly different ((mean ± SEM: 8853.4 ± 2569.5/*μ*L vs. 12962.4 ± 2560.2/*μ*L) and (median (IQR): 3602.0/*μ*L (1765.0–12455.0/*μ*L) vs. 4710.5/*μ*L (1934.3–15256.0/*μ*L), respectively. The PDMPs level on 30 days after discharge between subjects with 30-day MACE and those without was also not significantly different ((mean ± SEM: 18263.5 ± 6056.8/*μ*L vs. 19921.2 ± 3274.9/*μ*L) and (median (IQR): 8020.0/*μ*L (1612.0–15710.0/*μ*L) vs. 9684.5/*μ*L (1473.5–30976.8/*μ*L), respectively. Among subjects with 30-day MACE, the increased PDMPs level from the day of admission to 30 days after discharge was statistically significant (*p*=0.010, Wilcoxon signed rank test). Among subjects without 30-day MACE, the increased PDMP value from the day of admission to 30 days after discharge was not statistically significant (*p*=0.244, Wilcoxon signed rank test).  Figure 3 shows the PDMPs level among subjects in association with 30-day MACE.

### 4.5. Increased PDMPs Level Associated with 30-Day, 90-Day, and 1-Year MACE

**Figure 4:**
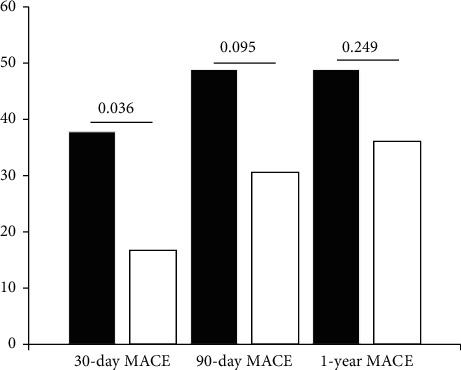
The occurrence of MACE between subjects with an increased PDMPs level (black bars) and decreased PDMPs level (white bars) in 30 days after discharge (37.8% vs. 16.7%, *p*=0.036), 90 days after discharge (48.9% vs. 30.6%, *p*=0.095), and 1 year after discharge (48.9% vs. 36.1%, *p*=0.249).

As many as 45 subjects had an increased PDMPs level in the 30-day postdischarge measurement as compared with the measurement on the day of admission. The comparison of characteristics between subjects who had an increased PDMPs level in 30 days after discharge and those with a decreased PDMPs level (*n* = 36) is shown in Table 3. Among subjects with an increased PDMPs level, there was a significantly higher 30-day MACE occurrence as compared to subjects with a decreased PDMPs level 17(37.8%) vs. 6 (16.7%, *p*=0.036). In the 90-day and 1-year follow-up, there was a trend toward higher MACE occurrence in subjects with an increased PDMPs level as compared to subjects with a decreased PDMPs level in 90 days after discharge (48.9% vs. 30.6%, *p*=0.095) and 1 year after discharge (48.9% vs. 36.1%, *p*=0.249) (Figure 4).

## 5. Discussion

The results of our study demonstrated that the PDMPs level was increased at 30 days after acute STEMI as compared with on-admission acute STEMI. Our study indicated that the persistent increase of the PDMPs level at 30 days after acute STEMI was associated with MACE within 30 days after discharge. The trend toward increased MACE was extended during the 90-day follow-up after the acute STEMI event, in subjects with an increased PDMPs level.

Post-myocardial infarction MACE following acute STEMI is a devastating result which can lead to considerable morbidity and mortality. Currently, the timely revascularization intervention has reduced the fatal MACE rate; however, morbidities, such as left ventricle dysfunction and heart failure, endure. The inflammation states during and following acute STEMI, which are generated and activated by damaged cardiomyocytes, might have an unfavorable outcome on left ventricular remodeling, atherosclerosis, and the tendency of recurrent thrombosis [12]. The progressive left ventricle dilation and dysfunction following STEMI is due to the remodeling response of noninfarcted cardiomyocytes, along with the infarcted scars and altered pressure and volume of the left ventricle [13]. The present study indicated that the most common MACEs during and following STEMI were left ventricle dysfunction and heart failure.

The PDMPs are small extracellular vesicles with a diameter between 0.1 and 1 *μ*m which are derived from platelet plasma membranes [14]. They are released in response to various stimuli on platelets, mainly platelet activation and inflammation [15,16]. The cytoplasmic contents and membrane proteins from platelets are owned by PDMPs; therefore, they have the capability of cell-to-cell interaction similar to their parental cells [15]. The PDMPs release thromboxane A2 and participate in platelet aggregation and thrombus formation [16]. They also interact with leukocytes which intensify the inflammatory state [17]. In acute STEMI, platelet activation and inflammatory response induce the shedding of platelet plasma membranes and PDMPs release. It subsequently promotes coronary vasoconstriction, perpetuating thrombus occlusion and inducing leukocyte recruitment in infarcted cardiomyocytes. Translating into clinical scenarios, these pathomechanisms affect the adverse outcomes in STEMI, such as left ventricle dysfunction, acute heart failure, fatal arrhythmia, and reinfarction. The present study indicated that subjects with in-hospital MACE had the tendency toward a lower number of circulating PDMPs as compared with subjects without MACE.

In the coronary circulation, PDMPs are abundantly found in the occluded coronary segments in STEMI [4,18]. The intracoronary PDMPs levels were higher than the circulating levels in acute STEMI [19]. In the occluded coronary segment, high shear stress excites further platelet deposition and thrombus growth in which PDMPs bind to adhered platelets and enhance platelet and fibrin deposition [19]. The percutaneous coronary intervention with stent implantation and plaque rupture stimulate an increased PDMPs release into the coronary circulation [17,18]. Studies indicated that circulating PDMP levels were lower in STEMI than NSTEMI, and long-term measurement indicated that CD62P[+]-PDMPs were slightly decreased within 90 days after acute STEMI [20]. In contrast, the present study indicated that, overall, the PDMPs level was significantly increased in 30 days after the acute STEMI event. More than half of the subjects had an increased PDMPs level at 30 days after discharge. This significant increase of the PDMPs level was associated with a higher occurrence of MACE in 30 days. Further follow-up until 2 years from the event showed that the PDMPs level had a persistent increasing trend [20]. In our observations, subjects with an increased PDMPs level in 30 days after acute STEMI had significantly higher MACE in the 30-day follow-up, and the trend persisted at the 90-day follow-up.

The lack of decline of the circulating PDMPs level after acute STEMI overtime indicates that the circulating PDMPs do not reflect thrombus burden and lesion on the coronary artery [20]. There is a significant increase in PDMPs release in coronary circulation after plaque rupture and percutaneous intervention, which do not extend into the systemic circulation [19,21]. The persistence of the circulating PDMPs level after the acute STEMI event may have different dynamic levels as compared with PDMP levels in the coronary circulation, based on the surface marker determination. Coronary artery plaque rupture and platelet activation are the earliest sources of the PDMPs level in acute STEMI events. In our study, there was no significant difference in the circulating PDMPs level between subjects who underwent primary PCI and in those we performed fibrinolysis.

In the present study, the circulating PDMPs level in the acute STEMI was not associated with the in-hospital and after-discharge MACE. However, the persistent increase of the PDMPs level from the day of admission to 30 days after the acute STEMI event was associated with greater MACE. In CD62P(+)-PDMPs, the microparticles can adhere to its ligand P-selectin glycoprotein 1 [PSGL-1], which induces cellular signaling in neutrophils and monocytes and accumulation of tissue factors in the occluded thrombi [20]. The impact of this interaction is an aggravated inflammatory and procoagulant state [20]. The CD62P(+)-PDMPs had the highest expression of tissue factor which can attach to protease-activated receptors and induce cellular signaling causing inflammation [22]. These mechanisms might be explained by the observable fact that PDMPs expressing tissue factor are tightly adhered to the vessel walls and induce inflammation [22]. In accordance with this hypothesis, lower concentrations of circulating CD62P(+)-PDMPs tended to be associated with worse clinical outcomes. These results pointed to the complexity of evaluating PDMPs in circulating plasma as a biomarker for the risk of clinical outcomes. Our study indicated a tendency toward a decreased CD62P(+)-PDMPs level and worse angiographic characteristics in subjects who experienced in-hospital MACE and 30-day MACE.

Our study findings have several clinical implications that are attributed to the measurement and function of PDMPs. First, the earliest treatment strategy for STEMI should consider the optimal use of medication to reduced PDMPs release, for example, high-dose antiplatelets and/or anticoagulants as complements for revascularization [23]. Second, the optimal antiplatelet therapy in the early after acute STEMI should be emphasized and applied. The best combination of antiplatelets and, sometimes, with oral anticoagulant should be individualized based on the propensity of the thromboembolic state. Finally, the measurement of a specific subtype of PDMPs as a marker of thromboembolic and hypercoagulability states in circulation should be considered in patients with STEMI as a prognostic biomarker [24].

In conclusion, the PDMPs level was increased from the day of admission to 30 days after discharge in patients with STEMI. The PDMPs level was significantly increased from the day of admission to 30 days after discharge in subjects who experienced MACE within 30 days after discharge. The persistent increase of the PDMPs level in 30 days after acute STEMI was associated with MACE within 30 days after discharge and trended toward increased MACE which extended during the patients' 90-day and 1-year follow-up.

## Figures and Tables

**Table 1:** Characteristics of subjects with STEMI and their comparison based on the occurrence of in-hospital MACE.

Characteristics	All subjects *n* = 101	In-hospital MACE (+) *n* = 17	In-hospital MACE (−) *n* = 84	*p* value
Gender (male), *n* (%)	95 (94.1)	17 (100)	78 (92.9)	0.321
Age (years), mean ± SD	54.5 ± 8.9	55.6 ± 7.9	54.3 ± 9.2	0.594
Dyslipidemia, *n*(%)	24 (23.8)	6 (35.3)	18 (21.4)	0.221
Diabetes mellitus, *n* (%)	24(23.8)	5 (29.4)	19 (22.6)	0.548
Ischemic heart disease, *n* (%)	22 (21.8)	3 (17.6)	19 (22.6)	0.651
Hypertension, *n* (%)	45 (44.6)	11 (64.7)	34 (40.5)	0.067
Current smoker, *n* (%)	62 (61.4)	11 (64.7)	51 (60.7)	0.693
Onset (hours), median (IQR)	4.5(3.0–7.0)	5 (3.0–7.2)	4.5 (3.0–7.0)	0.643
Systolic BP (mmHg), mean ± SD	135.3 ± 25.1	130.2 ± 18.8	136.3 ± 26.1	0.368
Diastolic BP (mmHg), mean ± SD	83.2 ± 13.8	82.0 ± 13.8	83.4 ± 13.9	0.702
Heart rate (x/min), mean ± SD	79.2 ± 17.6	91.1 ± 19.7	76.8 ± 16.2	0.002
Anterior STEMI, *n* (%)	55 (54.5)	12 (70.6)	43 (51.2)	0.328
Killip class ≥2	14 (13.9)	14 (82.6)	0 (0)	<0.001
Revascularization^*∗*^				0.123
Primary PCI	52 (61.2)	11 (64.7)	41 (48.8)	
Fibrinolysis	33 (38.8)	2 (11.8)	31 (36.9)	
Vessel disease^*∗∗*^				0.726
1 vessel disease	25 (38.5)	4 (23.5)	21 (25.0)	
2 vessel disease	19 (29.2)	4 (23.5)	15 (17.9)	
3 vessel disease	18 (27.7)	4 (23.5)	14 (16.7)	
Any + left main disease	3 (4.6)	1 (5.9)	2 (2.4)	
Culprit vessel^*∗∗*^				0.164
Left anterior descendent	41 (63.1)	11 (64.7)	30 (35.7)	
Right coronary artery	22 (33.8)	2 (11.8)	20 (23.8)	
Left circumflexus	2 (3.1)	0 (0)	2 (2.4)	
Intervention^*∗∗*^				0.332
Drug-eluting stent	62 (95.4)	13 (76.5)	49 (58.3)	
Bare metal stent	3 (4.6)	0 (0)	3 (3.5)	
Haemoglobin (g/dL)	13.9 ± 1.6	13.6 ± 1.6	14.0 ± 1.6	0.299
Leucocytes (×10^3^/mm^3^)	13.4 ± 3.6	14.7 ± 5.2	13.2 ± 3.2	0.239
Platelets (×10^3^/mm^3^)	268.9 ± 82.3	273.6 ± 75.8	267.9 ± 83.9	0.796
Creatinine (mg/dL)	1.2 ± 0.4	1.4 ± 0.4	1.2 ± 0.4	0.034
Urea nitrogen (mg/dL)	15.5 ± 6.8	18.0 ± 9.9	15.0 ± 5.9	0.240
Random glucose (mg/dL)	168.2 ± 77.4	174.5 ± 91.6	166.9 ± 74.8	0.722
Clopidogrel	101 (100)	17 (100)	84 (100)	NA
Acetylsalicylic acid	101 (100)	17 (100)	84 (100)	NA
ACEI/ARB	68 (67.3)	14 (82.4)	54 (64.3)	0.147
Beta blockers	61 (60.4)	11 (64.7)	50 (59.5)	0.690
Furosemide	16 (15.8)	11 (64.7)	5 (6.0)	<0.001
Heparin	64 (63.4)	10 (58.8)	54 (53.4)	0.670

^*∗*^ *n* = 85 subjects; ^*∗∗*^*n* = 65 subjects. BP: blood pressure; STEMI: ST-elevation myocardial infarction; ACEI: angiotensin-converting enzyme inhibitors; ARB: angiotensin-receptor blocker: NA: not applicable.

**Table 2:** Characteristics of subjects with STEMI and their comparison based on the occurrence of 30-day postdischarge MACE.

Characteristics	All subjects (*n* = 81)	30-day MACE (+) (*n* = 23)	30-day MACE (−) (*n* = 58)	*p* value
Gender (male), *n* (%)	75 (92.6)	21 (91.3)	54 (93.1)	0.551
Age (years), mean ± SD	54.4 ± 9.1	55.6 ± 7.9	54.3 ± 9.2	0.594
Dyslipidemia, *n* (%)	22 (27.2)	6 (26.1)	16 (27.6)	0.891
Diabetes mellitus, *n* (%)	18(22.2)	7 (30.4)	11 (19.0)	0.263
Ischemic heart disease, *n* (%)	16 (19.8)	4 (17.4)	12 (20.7)	0.501
Hypertension, *n* (%)	36 (44.4)	14 (60.9)	22 (37.9)	0.061
Current smoker, *n* (%)	50 (61.7)	14 (60.9)	36 (62.1)	0.251
Onset (hours), median (IQR)	4.3(3.0–7.0)	5 (3.0–7.2)	4.5 (3.0–7.0)	0.643
Systolic BP (mmHg), mean ± SD	135.3 ± 25.3	130.2 ± 18.8	136.3 ± 26.1	0.368
Diastolic BP (mmHg), mean ± SD	83.7 ± 13.7	82.0 ± 13.8	83.4 ± 13.9	0.702
Heart rate (x/min), mean ± SD	79.1 ± 18.2	91.1 ± 19.7	76.8 ± 16.2	0.002
Anterior STEMI, *n* (%)	48 (59.3)	15 (65.2)	33 (56.9)	0.492
Killip class ≥2	11 (13.6)	11 (47.8)	0 (0)	<0.001
Revascularization^*∗*^				0.090
Primary PCI	44 (63.8)	16 (84.2)	28 (56.0)	
Fibrinolysis	25 (36.2)	3 (15.8)	22 (44.0)	
Vessel disease^*∗∗*^				0.544
1 vessel disease	22 (41.5)	6 (31.6)	16 (47.1)	
2 vessel disease	16 (30.1)	6 (31.6)	10 (29.4)	
3 vessel disease	12 (22.6)	5 (26.3)	7 (20.6)	
Any + left main disease	3 (5.6)	2 (10.5)	1 (2.9)	
Culprit vessel^*∗∗*^				0.261
Left anterior descendent	35 (66.0)	15 (78.9)	20 (58.8)	
Right coronary artery	16 (30.1)	4 (21.1)	12 (35.3)	
Left circumflexus	2 (2.5)	0 (0)	2 (5.9)	
Intervention^*∗∗*^				0.255
Drug-eluting stent	50 (94.3)	19 (100.0)	31 (91.2)	
Bare metal stent	3 (5.7)	0 (0)	3 (8.8)	
Haemoglobin (g/dL)	13.9 ± 1.7	13.7 ± 1.7	14.0 ± 1.7	0.401
Leucocytes (×10^3^/mm^3^)	13.3 ± 3.5	12.9 ± 4.1	13.5 ± 3.3	0.553
Platelets (×10^3^/mm^3^)	265.9 ± 80.1	265.3 ± 74.4	266.2 ± 82.8	0.964
Creatinine (mg/dL)	1.2 ± 0.4	1.3 ± 0.5	1.2 ± 0.4	0.497
Urea nitrogen (mg/dL)	15.1 ± 6.2	15.5 ± 7.8	14.9 ± 5.5	0.700
Random glucose (mg/dL)	166.6 ± 73.4	185.9 ± 96.3	158.4 ± 60.5	0.216
Clopidogrel	81 (100)	17 (100)	84 (100)	NA
Acetylsalicylic acid	81 (100)	17 (100)	84 (100)	NA
ACEI/ARB	54 (66.7)	17 (73.9)	37 (63.8)	0.384
Beta blockers	51 (63.0)	18 (78.3)	33 (56.9)	0.073
Furosemide	14 (17.3)	9 (39.1)	5 (8.6)	0.001
Heparin	53 (65.4)	15 (65.2)	38 (65.5)	0.980

^*∗*^ *n* = 69 subjects ^*∗∗*^*n* = 53 subjects. BP: blood pressure; STEMI: ST-elevation myocardial infarction; ACEI: angiotensin-converting enzyme inhibitors; ARB: angiotensin-receptor blocker; NA: not applicable.

**Table 3:** Characteristics of subjects based on the PDMPs level measured in 30 days after discharge.

Characteristics	PDMPs increased (*n* = 45)	PDMPs decreased (*n* = 36)	*p* value
Gender (male), *n* (%)	41 (91.1)	34 (94.4)	0.450
Age (years), mean ± SD	54.6 ± 9.3	54.1 ± 8.9	0.795
Dyslipidemia, *n* (%)	11 (24.4)	11 (30.6)	0.539
Diabetes mellitus, *n* (%)	8 (17.8)	10 (27.8)	0.282
Ischemic heart disease, *n* (%)	6 (13.3)	10 (27.8)	0.105
Hypertension, *n* (%)	19 (42.2)	17 (47.2)	0.653
Current smoker, *n* (%)	27 (60.0)	23 (63.9)	0.709
Onset (hours), median (Q1-Q3)	6.0 ± 4.6	5.1 ± 3.7	0.365
Systolic BP (mmHg), mean ± SD	129.8 ± 20.3	142.1 ± 29.4	0.028
Diastolic BP (mmHg), mean ± SD	80.6 ± 11.9	87.5 ± 15.0	0.025
Heart rate (x/min), mean ± SD	78.0 ± 16.2	80.4 ± 20.6	0.573
Anterior STEMI, *n* (%)	23 (51.1)	25 (69.4)	0.095
Killip class ≥2	5 (11.1)	6 (16.7)	0.468
Revascularization^*∗*^			0.689
Primary PCI	24 (64.8)	20 (62.5)	
Fibrinolysis	13 (48.1)	12 (37.5)	
Vessel disease^*∗∗*^			0.208
1 vessel disease	11 (34.4)	11 (52.4)	
2 vessel disease	10 (31.3)	6 (28.6)	
3 vessel disease	10 (31.3)	2 (9.5)	
Any + left main disease	1 (3.1)	2 (9.5)	
Culprit vessel^*∗∗*^			0.296
Left anterior descendent	19 (59.4)	16 (76.2)	
Right coronary artery	11 (34.4)	12 (23.8)	
Left circumflexus	2 (6.3)	0 (0)	
Intervention^*∗∗*^			0.303
Drug-eluting stent	31 (96.9)	19 (90.5)	
Bare metal stent	1 (3.1)	2 (9.5)	
Haemoglobin (g/dL)	14.2 ± 1.7	13.6 ± 1.6	0.084
Leucocytes (×10^3^/mm^3^)	12.8 ± 3.2	13.9 ± 3.8	0.150
Platelets (×10^3^/mm^3^)	269.9 ± 94.6	260.9 ± 57.9	0.620
Creatinine (mg/dL)	1.1 ± 0.4	1.3 ± 0.5	0.019
Urea nitrogen (mg/dL)	13.8 ± 4.4	16.8 ± 7.7	0.040
Random glucose (mg/dL)	170.0 ± 81.3	162.4 ± 62.9	0.653
Clopidogrel	45 (100)	36 (100)	NA
Acetylsalicylic acid	45 (100)	36 (100)	NA
ACEI/ARB	28 (62.2)	26 (72.2)	0.343
Beta blockers	28 (62.2)	23 (63.9)	0.877
Furosemide	8 (17.8)	6 (16.7)	0.895
Heparin	32 (71.1)	21 (58.3)	0.230

^*∗*^ *n* = 69, ^*∗∗*^*n* = 53. BP: blood pressure; STEMI: ST-elevation myocardial infarction; ACEI: angiotensin-converting enzyme inhibitors; ARB: angiotensin-receptor blocker; PDMPs: platelet-derived microparticles; NA: not applicable.

## Data Availability

The original coded data used to support the findings of this study were supplied by Anggoro Budi Hartopo MD, PhD, under license and so cannot be made freely available. Requests for access to these data should be made to Anggoro Budi Hartopo MD, PhD (a_bhartopo@ugm.ac.id).
